# Association of *ACE2* Polymorphisms and Derived Haplotypes With Obesity and Hyperlipidemia in Female Spanish Adolescents

**DOI:** 10.3389/fcvm.2022.888830

**Published:** 2022-05-02

**Authors:** Jairo Lumpuy-Castillo, Claudia Vales-Villamarín, Ignacio Mahíllo-Fernández, Iris Pérez-Nadador, Leandro Soriano-Guillén, Oscar Lorenzo, Carmen Garcés

**Affiliations:** ^1^Laboratory of Diabetes and Vascular Pathology, IIS-Fundación Jiménez Díaz, Universidad Autónoma, Madrid, Spain; ^2^Spanish Biomedical Research Centre on Diabetes and Associated Metabolic Disorders (CIBERDEM) Network, Madrid, Spain; ^3^Lipid Laboratory, IIS-Fundación Jiménez Díaz, Universidad Autónoma, Madrid, Spain; ^4^Epidemiological Research Unit, IIS-Fundación Jiménez Díaz, Madrid, Spain; ^5^Department of Pediatrics, IIS-Fundación Jiménez Díaz, Universidad Autónoma, Madrid, Spain

**Keywords:** ACE2, SNP, haplotype, cardiovascular, obesity, hyperlipidemia

## Abstract

**Background:**

In the cardiovascular (CV) system, overactivation of the angiotensin converting enzyme (ACE) may trigger deleterious responses derived from angiotensin (Ang)-II, which can be attenuated by stimulation of ACE2 and subsequent Ang-(1-7) metabolite. However, *ACE2* exhibits a high degree of genetic polymorphism that may affect its structure and stability, interfering with these cardioprotective actions. The aim of this study was to analyse the relationship of *ACE2* polymorphisms with cardiovascular risk factors in children.

**Methodology:**

Five *ACE2*-single nucleotide polymorphisms (SNP), rs4646188, rs2158083, rs233575, rs879922, and rs2074192, previously related to CV risk factors, were analyzed in a representative sample of 12–16-year-old children and tested for their potential association with anthropometric parameters, insulin levels and the lipid profile.

**Results:**

Girls (*N* = 461) exhibited lower rates of overweight, obesity, blood pressure, and glycemia than boys (*N* = 412), though increased plasma lipids. The triglycerides (TG)/HDL-C ratio was, however, lower in females. Interestingly, only in girls, the occurrence of overweight/obesity was associated with the SNPs rs879922 [OR 1.67 (1.02–2.75)], rs233575 [OR 1.98 (1.21- 3.22)] and rs2158083 [OR 1.67 (1.04–2.68)]. Also, TG levels were linked to the rs879922, rs233575, and rs2158083 SNPs, and the TG/HDL-C ratio was associated with rs879922 and rs233575. Levels of TC and LDL-C were associated with rs2074192 and rs2158083. Furthermore, the established cut-off level for TG ≥ 90 mg/dL was related to rs879922 [OR 1.78 (1.06–2.96)], rs2158083 [OR 1.75 (1.08–2.82)], and rs233575 [OR 1.62 (1.00–2.61)]. The cut-off level for TC ≥ 170 mg/dL was associated with rs2074192 OR 1.54 (1.04–2.28) and rs2158083 [OR 1.53 (1.04–2.25)]. Additionally, the haplotype (C-G-C) derived from rs879922-rs2158083-rs233575 was related to higher prevalence of overweight/obesity and TG elevation.

**Conclusion:**

The expression and activity of ACE2 may be essential for CV homeostasis. Interestingly, the *ACE2*-SNPs rs879922, rs233575, rs2158083 and rs2074192, and the haplotype (C-G-C) of the three former could induce vulnerability to obesity and hyperlipidemia in women. Thus, these SNPs might be used as predictive biomarkers for CV diseases and as molecular targets for CV therapy.

## Introduction

The Angiotensin converting enzyme-2 (ACE2) is a significant regulator of the Renin-Angiotensin-Aldosterone system (RAAS), which plays key roles in the control of cardiovascular (CV) system (1). In the canonical pathway, the RAAS precursor, angiotensinogen, is degraded to the octapeptide angiotensin-II (Ang-II) by consecutive digestions of renin and angiotensin convertase enzyme (ACE). Ang-II could be then converted by aminopeptidase-A to Ang-III, which conserves similar proprieties mostly mediated by two distinct G protein-coupled receptors named AT1R and AT2R (2). Overactivation of the ACE/Ang-II/AT1R pathway has been associated with several CV pathologies, including hypertension, heart failure, vascular inflammation and remodeling, coagulation, and atherosclerosis (3). However, activation of AT2R can trigger anti-inflammation and vasodilation. In this regard, the stimulation of the non-canonical axis of the RAAS may compensate ACE/Ang-II/AT1R actions. The mono-carboxypeptidase ACE2 can convert Ang-II into angiotensin 1–7 [Ang (1-7)], which binds to the Mas receptor (MasR) (1). The ACE2/Ang (1-7)/MasR axis can reduce blood pressure and CV hypertrophy and fibrosis, stimulating vasoactive prostaglandins and lessening redox imbalance (4). Ang (1-7) also protects ACE-degradation of bradykinins and ameliorates inflammatory responses, and vascular permeability (1). Moreover, ACE2 also degrades Ang l into Ang (1–9), decreasing Ang-II levels. Given the opposite effects of Ang (1-7) and Ang-II in the CV homeostasis, it might be imperative to maintain a minimum ACE2 level or activity.

**Figure 1:**
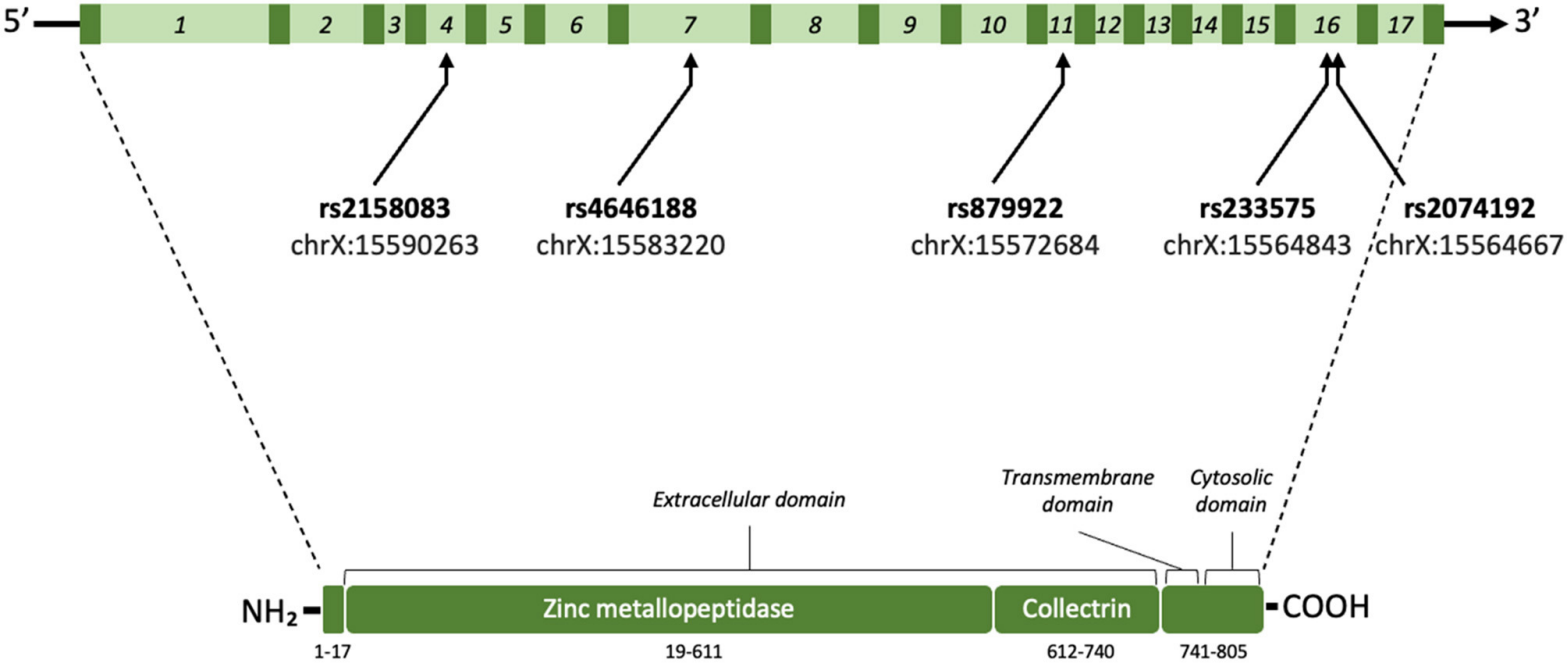
Schematic illustration of ACE2 gene and protein. The homo sapiens *ACE2* gene (3,339 base pairs) is located on the chromosome X (Xp22.2; nucleotides 15,494,402–15,602,148; GRCh38.hg38 version). *ACE2* comprises 18 exons (dark green) and 17 introns (light green; 1–17 numbers) where the rs4646188, rs879922, rs233575, rs2074192, and rs2158083 SNPs can be found. After mRNA processing and splicing, the full length ACE2 protein includes 805 amino acids with different regions and domains [signal peptide (1–17 aa), Zinc-binding metalloproteinase (19–611 aa), collectrin-like domain (612–740 aa), and C-terminal transmembrane anchoring region (741–805 aa)] (https://www.uniprot.org/uniprot/Q9BYF1).

Interestingly, the *ACE2* gene is highly conserved in mammals and expressed in testes, renal and CV system, and especially in gastrointestinal tissues (5). It encodes a type I membrane-bounding glycoprotein composed by 805 amino acids divided in three functional domains including a C-terminal transmembrane anchoring region, a N-terminal signal peptide motif, and an HEXXH zinc-binding metalloprotease region (Figure 1). *ACE2* maps in chromosome Xp22 and contain 17 introns and 18 exons, where a high degree of genetic polymorphism can be found (6). There are at least 510 valid variants in *ACE2* (mostly intronic regions) (7), and some single nucleotide polymorphisms (SNP) may affect ACE2 structure and stability, interfering with its cardioprotective functions. In adults with different gender, ethnic and presence of pre-existing CV disease, the rs4646188, rs2158083, rs233575, rs879922, and rs2074192 SNPs (Figure 1) have been related to hypertension, ventricular hypertrophy, or type-2 diabetes mellitus (T2DM), and increased plasma total cholesterol (TC), triglycerides (TG), low-density lipoprotein-cholesterol (LDL-C), or reduced high-density lipoprotein-cholesterol (HDL-C) and Ang (1-7) (8–11). In children or adolescent cohorts, where existence of confusing factors (i.e., epigenetic alterations) is lower, boys but not girls showed association of rs2158083, rs233575 and rs2074192 with hypertension (12, 13). Nowadays, regulation of the ACE2 expression and activity has become essential for Coronavirus disease (COVID)-19 subjects, particularly for those with cardiovascular complications (6, 14). Thus, further studies analyzing *ACE2-*SNPs and related haplotypes in young populations, could suggest predictive markers for CV diseases and molecular targets for CV therapy.

In our study, we examine five *ACE2* SNPs in relationship with the presence of overweight and obesity in a representative sample of 12-to 16-year-old children, and we assess the potential association of the polymorphisms with the lipid profile and insulin levels.

## Methodology

### Subjects

The present study comprised 873 adolescents (11–17 years old) recruited in the “Four Provinces study” during 2004–08 in whom information on biochemical variables and DNA samples were available (15). This cross-sectional study was designed to analyze CV risk factors in general population of adolescents from Spain (461 girls and 412 boys). Children suffering from any endocrine, metabolic, hepatic, or renal disorder were excluded from the study. Specialized physicians and nurses oversaw the anthropometric characterization and blood extractions. The investigation fulfills the principles contained in the Declaration of Helsinki and subsequent reviews, as well as the prevailing Spanish legislation on clinical research in human subjects. Parents were required to sign a written consent form allowing their children to participate. The study protocol was approved by the Ethics Committee of Clinical Investigation of the Fundación Jiménez Díaz (Code number: PIC016-2019 FJD).

### Variables of the Study

#### Anthropometric and Blood Pressure Measurements

The weight and height were taken in barefoot children, wearing light clothing. Both weight and height were approached to the nearest 0.1 unit (kg or cm, respectively) and were used to estimate the body mass index (BMI), as kilograms divided by height in squared meters (kg/m^2^). The age- and sex-specific cut-off points of BMI for overweight and obesity were established according to Cole et al. (16). In addition, the blood pressure (systolic and diastolic) was quantified in subjects as average of two measurements in the right arm by using a mercury sphygmomanometer and after 15 min resting in seated position.

#### Plasma Parameters

Blood samples were obtained from subjects in the morning after a 12-h fasting period by using EDTA-collection tubes (Vacutainer, BD). Plasma was separated by centrifuging blood at 1,500 g at 4°C for 20 min. The upper fraction was stored at −80°C until use. Total cholesterol and triglycerides were enzymatically quantified in a RA-1000 Autoanalyzer (Technicon, Luton, UK). HDL-C concentrations were measured after precipitation of apo B-containing lipoproteins with phosphotungstic acid and Mg^++^ (Boehringer Mannheim, Baden-Wurttemberg, Germany). LDL-C levels were calculated according to Friedewald's formula. Also, the glucose concentration was evaluated by the glucose oxidase method, and insulin levels were achieved by using a RIA commercial kit (BI-Insulin IRMA, Bio-Rad, France). To estimate insulin resistance, the homeostatic model assessment of insulin resistance (HOMA-IR) index was calculated following formula: fasting insulin (μU/mL) x fasting glucose (mmol/liter)/22.5.

#### Single Nucleotide Polymorphism (SNPs) Determinations

Genomic DNA was also obtained from blood samples. After plasma separation, the interphasic fraction (leukocytes) was used to isolate total DNA by adaptation of a classic procedure, which involves salting out of the cellular proteins by dehydration and precipitation with a saturated NaCl solution (17). The quantity and quality of DNA was assessed by UV-spectrophotometry using the Nanodrop spectrophotometer ND-1000. Five SNPs located in intronic sequences of the *ACE2* gene (A/G [rs4646188], G/C [rs879922], A/G [rs233575], C/T [rs2074192] and T/C [rs2158083]) were genotyped by using predesigned TaqMan® SNP Genotyping Assays from Applied Biosystems (C_32336232_10, C_8877953_10, C_2260459_10, C_16163821_10 and C_16141434_10, respectively). A StepOnePlus™ Real-Time PCR System (Applied Biosystems) was used for allelic discrimination. PCR was performed with a mixture of 10 ng of genomic DNA, TaqMan® SNP Genotyping Assay (20X), and TaqPath™ ProAmp™ Master Mix (Applied Biosystems). Samples were cycled under the recommended conditions: 95 °C for 10 min, 95 °C for 15 s and 60 °C for 1 min, repeated over 40 cycles. The data presented in the study are deposited in the dbSNP repository, submission batch ID 1063361 (dbSNP Build ID: B156). The genotyping data were upload in a public repository (accession number).

#### Haplotype Analysis

The five SNPs (rs4646188, rs879922, rs233575, rs2074192, and rs2158083) were selected to construct haplotypes. Linear generalized models were used to compare outcomes levels between haplotypes. The comparisons were summarized by coefficients and odds ratios for continuous and categorical outcomes respectively, and with 95% confidence intervals and p-values. These analyses were performed using the haplo.glm function from the haplo.stats R package. Haplotypes with frequencies less than 5% were included in the category “other”.

### Statistical Analysis

Qualitative variables were included as absolute and relative frequencies. Associations between qualitative variables were studied by the chi-square test and odds ratio. On the other hand, normality of quantitative variables was analyzed by the Kolmogorov–Smirnov test. On one hand, variables with normal distribution (age and HDL-C) were compared using a Student's t-test and ANOVA one way, followed by Tukey (assuming equal variances) or Games-Howell (equal variances not assumed) *post-hoc* test. Also, they were summarized as mean values and 95% confidence interval (CI). Some variables with non-normal distribution were normalized by log base-10 transformation [systolic (SBP) and diastolic (DBP) blood pressures, TC, TG, LDL-C, TG/HDL] and were expressed as geometric mean values and 95% confidence interval. Other variables with non-normal distribution (glucose, insulin, and HOMA-IR) were compared using the Mann–Whitney *U* test and Kruskal-Wallis test followed by Dunn's (*post-hoc*). These variables were summarized by median and interquartile range. The associations of haplotypes with anthropometric, blood pressure and plasma parameters were analyzed by regression models. For continuous variables, we used linear models, whereas dichotomous coded variables were examined by logistic models. The former were summarized by coefficients (coef.) while the later were summarized by odds ratio (OR). Also, a 95% CI and p-values were reported. Statistical analyses were performed using the statistical language R version 4.0.5 (R Foundation for Statistical Computing, Vienna, Austria).

## Results

### Characterization of the Population

We analyzed a population of 873 adolescents aged 13.9 years with a mean BMI of 21.5 kg/m^2^. In the study, we found that 71.8 percent of children were normo-weight, whereas 28.2 percent presented an excessive weight (Table 1). The prevalence of obesity in girls and boys was 4.3 and 7.6 %, respectively. The mean BMI Z-score was 0.00 (−0.067–0.067) for the global population, and −0.018 (−0.10–0.07) and 0.021 (−0.08–0.12), for girls and boys, respectively (*p* = 0.569). Also, according to the latest clinical guides of the American College of Cardiology/American Heart Association/American Diabetes Association for pediatric population (18, 19), they were, on average, in the normotensive range and showed unaltered levels of the glycemic and lipid profiles (Table 1). However, further analysis pointed out significant differences between females and males. Females (52.8%) exhibited lower rates of overweight and obesity, SBP and glycemia, but higher levels of TC, LDL-C, and HDL-C than boys (Table 1). The ratio of TG and HDL-C, as a predictor of metabolic syndrome and CV diseases (20), was significantly lower in female individuals.

**Table 1:** Anthropometric and biochemical characterization of a Spanish population of adolescents.

**Variables**	**Global (*N =* 873)**	**Female (*N =* 461)**	**Male (*N =* 412)**	***P*** **-value**
Age (years)	13.9 (13.8–13.9)	13.9 (13.7–14.0)	13.9 (13.7–14.0)	0.873
BMI (kg/m^2^)	21.5 (21.3–21.8)	21.5 (21.1–21.8)	21.6 (21.2–21.9)	0.720
NW % (n)	71.8 (625)	**75.7 (349)**	**67.3 (276)**	**0.006**
OW + Obesity % (n)	28.2 (246)	**24.3 (112)**	**32.7 (134)**	**0.006**
SBP (mmHg)	113.9 (113.0–114.7)	**111.5 (110.3–112.6)**	**116.6 (115.3–117.8)**	**<0.001**
DBP (mmHg)	64.7 (64.0–65.4)	64.2 (63.2–65.2)	65.4 (64.4–66.3)	0.099
Glucose mg/dL	90.5 (10.6)	**89.0 (10.2)**	**92.3 (10.4)**	**<0.001**
Insulin (μU/mL)	7.44 (4.4)	7.54 (4.28)	7.32 (4.67)	0.249
HOMA–IR	1.65 (1.06)	1.64 (0.98)	1.66 (1.14)	0.867
TC (mg/dL)	163.9 (162.0–165.8)	**168.1 (165.6–170.7)**	**159.2 (156.6–161.9)**	**<0.001**
TG (mg/dL)	72.3 (70.5–74.0)	71.7 (69.4–74.0)	73.0 (70.2–75.8)	0.477
LDL–C (mg/dL)	94.9 (93.2–96.6)	**97.3 (94.9–99.7)**	**92.3 (89.9–94.7)**	**0.004**
HDL–C (mg/dL)	52.5 (51.5–53.5)	**54.6 (53.2–55.9)**	**50.2 (48.8–51.7)**	**<0.001**
TG/HDL–C ratio	1.43 (1.38–1.48)	**1.36 (1.30–1.43)**	**1.51 (1.43–1.60)**	**0.004**

*Data are expressed as %, median and interquartile range (IR), mean or geometric mean (CI 95%). Variables with normal distribution (Age, BMI, SBP, DBP, TC, TG, LDL-C, HDL-C and TG/HDL-C ratio) were compared using t-Test, whereas variables with non-normal distribution (Glucose, Insulin, and HOMA-IR) were compared using the Mann–Whitney U test. Also, qualitative variables (NW and OW + Obesity) were studied by the Pearson's chi-square test. In bold, statistically significant data (p < 0.05)*.

*NW and OW, as normal weight and overweight, respectively. HOMA-IR, as Homeostatic Model Assessment of Insulin Resistance. TC, TG, LDL-C and HDL-c, for total cholesterol, triglycerides, low-density lipoprotein-cholesterol, and high-density lipoprotein-cholesterol, respectively*.

### Genotypic and Allelic Frequencies of *ACE2* SNPs

Five single nucleotide polymorphisms (SNPs) of the *ACE2* gene previously related to CV injuries (9, 10, 21) were analyzed in our study. The genotype frequencies of rs4646188, rs879922, rs233575, rs2074192, and rs2158083 were found in a Hardy's-Weinberg equilibrium. The prevalence of the minor alleles of the studied SNPs in girls and boys, respectively, were: (G) in rs4646188, 10.1% and 8.4%; (C) in rs879922, 42.4% and 39.3%; (G) in rs233575, 39.2% and 37.3%; (T) in rs2074192, 40.10% and 41.5%, and (C) in rs2158083, 38.45% and 36.3% (Table 2).

**Table 2:** Genotype and allele frequencies of *ACE2-*SNPs.

***ACE2*** **SNPs**	**Female**			**Male**	
	**Genotypes**	***n***	**Allele (%)**	***n***	**Allele (%)**
	**frequencies % (n)**			
rs4646188 A/G	AA: 80.7 (359)	445	A: 89.9	391	A: 91.6
	AG: 18.4 (82)		G: 10.1		G: 8.4
	GG: 0.9 (4)				
rs879922 G/C	GG: 33.2 (144)	434	G: 57.6	374	G: 60.7
	GC: 48.8 (212)		C: 42.4		C: 39.3
	CC: 18.0 (78)				
rs233575 A/G	AA: 36.8 (164)	446	A: 60.8	389	A: 62.7
	AG: 48.0 (214)		G: 39.2		G:37.3
	GG: 15.2 (68)				
rs2074192 C/T	CC: 37.0 (164)	443	C: 59.9	390	C: 58.5
	CT: 45.8 (203)		T: 40.10		T: 41.5
	TT: 17.2 (76)				
rs2158083 T/C	TT: 38.4 (171)	445	T: 61.55	391	T: 63.7
	TC: 46.3 (206)		C: 38.45		C: 36.3
	CC: 15.3 (68)				

*Five SNPs (rs4646188, rs879922, rs233575, rs2074192, and rs2158083) of the ACE2 gene, related to cardiovascular risk factors, were described in the population of adolescents as absolute and relative frequencies*.

### Association Between *ACE2* SNPs and Overweight and Obesity

We tested whether these five *ACE2* SNPs (rs4646188, rs879922, rs233575, rs2074192, and rs2158083) might associate with the anthropomorphic variables of the population. Remarkably, none of the SNPs were significantly related to the SBP or DBP, either in girls or boys (not shown). However, in females, the occurrence of overweight or obesity was found to be significantly associated with three of the studied SNPs (Table 3). In particular, the minor allele C of the rs879922 variant was present in 26.9% of overweight or obese females [OR 1.67 (95% CI: 1.02–2.75), *p* = 0.042]. Similarly, the G allele of rs233575 and the C allele of rs2158083 were present in 28.0% [OR 1.98 (1.21- 3.22), *p* = 0.006] and 27.0% [OR 1.67 (1.04–2.68), *p* = 0.032] of overweight or obese girls, respectively (Table 3). In contrast, the overweight or obesity condition was not related to any SNPs in the male population (Supplementary Table 1). Thus, the presence of rs879922, rs233575, and rs2158083 were associated with overweight and obesity only in adolescent females.

**Table 3:** Association of the *ACE2* rs4646188, rs879922, rs233575, rs2074192, and rs2158083 genotypes with overweight/obesity in female adolescents.

***ACE2*** **SNPs**	**NW % (*n*)**	**OW+**	**Pearson**	**OR (95%IC)**
		**Obesity % (*n*)**	**Chi-Square**	
rs4646188 A/G				
AA	76.6 (275)	23.4 (84)	0.841	-
AG + GG	75.6 (65)	24.4.0 (21)		
rs879922 C/G				
GG	81.9 (118)	18.1 (26)	**0.042**	**1.67 (1.02–2.75)**
GC + CC	73.1 (212)	26.9 (78)		
rs233575 A/G				
AA	83.5 (137)	16.5 (27)	**0.006**	**1.98 (1.21–3.22)**
AG + GG	72 (203)	28.0 (79)		
rs2074192 C/T				
CC	72.6 (119)	27.4 (45)	0.131	-
CT + TT	78.9 (220)	21.1 (59)		
rs2158083 T/C				
TT	81.9 (140)	18.1 (31)	**0.032**	**1.67 (1.04–2.68)**
TC + CC	73.0 (200)	27.0 (74)		

*The presence of rs4646188, rs879922, rs233575, rs2074192, and rs2158083 SNPs was analyzed in normo-weight (NW), and in overweight (OW)+obese girls. Data were described as absolute and relative frequencies (%) and associations between variables were analyzed by the Pearson's chi-square test, followed by logistic models. In bold, significant associations between variables (p < 0.05)*.

### Association Between *ACE2* SNPs and the Glucose and Lipid Profiles

Further potential relationships between the SNPs and biochemical variables were also assessed. Glycemic parameters such as glycemia, plasma insulin and the HOMA-IR were not significantly associated with the presence of any SNPs in any sex (not shown). Lipid parameters did not show any relationship with any SNPs in male adolescents. However, again in females, four out of five SNPs were related to the lipid profile. The highest levels of TG were significantly associated with the presence of heterozygous genotypes of rs879922 (*p* = 0.020), rs233575 (*p* = 0.017) and rs2158083 (*p* = 0.036) (Table 4). The TG/HDL-C ratio also linked with rs879922 and rs233575. In addition, the established cut-off levels of TG (over 90 mg/dL; 75th percentile) for adolescents (10–19 years-old) (18) were related to rs879922 [OR 1.78 (95% CI: 1.06-2.96)], rs2158083 [OR 1.75 (1.08–2.82)], and rs233575 [OR 1.61 (1.00–2.62)] (Figure 2A). Furthermore, the highest levels of TC and LDL-C were related to the heterozygous genotypes of rs2074192 (*p* = 0.003 and *p* = 0.03, respectively) and rs2158083 (*p* = 0.008 and *p* = 0.019, respectively) (Table 4). Similarly, the cut-off levels of TC (over 170 mg/dL; 75th percentile), but not that of LDL-C levels, for adolescents (10–19 years-old) (18) were associated with rs2074192 [OR 1.54 (95% CI: 1.04–2.28) and rs2158083 [OR 1.53 (1.04–2.25)] (Figure 2B). The HDL-C levels were, however, independent of these *ACE2* SNPs. Therefore, the presence of *ACE2* SNPs rs879922, rs233575 and rs2158083 may be useful to predict elevated levels of TG and TC in girls.

**Table 4:** Lipid levels by *ACE2* rs4646188, rs879922, rs233575, rs2074192, and rs2158083 genotypes in girls.

***ACE2*** **SNPs (*n*)**	**TC (mg/dL)**	**TG (mg/dL)**	**LDL–C (mg/dL)**	**HDL–C (mg/dL)**	**TG/HDL–C ratio**
rs879922 C/G					
GG (143)	166.4 (162.4–170.4)	66.9 (63.2–70.7)	95.4 (91.6–99.2)	55.72 (53.5–57.9)	1.2 (1.2–1.3)
GC (209)	171.5 (167.3–175.7)	74.0 (70.7–77.6)^*^	100.0 (96.3–103.9)	54.6 (52.6–56.6)	1.4 (1.3–1.5)^*^
CC (78)	163.2 (157.4–169.1)	71.4 (66.7–76.4)	93.0 (87.8–98.5)	53.96 (50.3–57.6)	1.4 (1.3–1.6)
rs233575 A/G					
AA (163)	166.3 (162.6–170.0)	67.6 (64.1–71.3)	95.4 (91.7–98.8)	55.6 (53.5–57.7)	1.3 (1.2–1.3)
AG (211)	171.0 (166.3–175.2)	74.7 (71.3–78.2)^*^	100.4 (96.5–104.2)	54.0 (52.0–55.9)	1.4 (1.4–1.5)^*^
GG (68)	163.8 (157.4–170.5)	71.8 (66.7–77.2)	93.5 (87.8–99.6)	53.9 (50.0–57.9)	1.4 (1.2–1.6)
rs2074192 C/T					
CC (164)	164.4 (159.9–168.9)	72.7 (69.2–76.2)	94.4 (90.4–98.6)	53.5 (51.2–55.8)	1.4 (1.3–1.5)
CT (200)	173.4 (169.6–177.3)^*^	72.2 (68.8–75.7)	101.1 (97.6–104.8)^*^	55.8 (53.7–57.8)	1.3 (1.3–1.4)
TT (75)	164.4 (159.4–169.6)#	66.9 (61.6–72.6)	95.3 (90.7–100.2)	54.0 (51.5–56.6)	1.3 (1.1–1.4)
rs2158083 T/C					
TT (170)	165.2 (161.4–169.0)	67.9 (64.7–71.4)	95.2 (91.9–98.7)	54.7 (52.7–56.6)	1.3 (1.2–1.4)
TC (203)	172.6 (168.6–176.8)^*^	74.3 (70.8–78.0)^*^	**101.0 (97.3–104.9)**	54.7 (52.6–56.8)	1.4 (1.3–1.5)
CC (68)	163.0 (156.7–169.6)^#^	72.0 (67.1–77.5)	92.6 (86.7–98.7)^#^	53.8 (50.2–57.4)	1.4 (1.2–1.6)

*The occurrence of rs4646188, rs879922, rs233575, rs2074192, and rs2158083 SNPs was studied in females classified according with the plasma levels of total cholesterol (TC), triglycerides (TG), low-density lipoprotein (LDL-C), high-density lipoprotein (HDL-C) and the TG/HDL-C ratio. Results are expressed as mean or geometric mean (CI 95%)*.

*Variables were compared by using an ANOVA one way test, followed by Tukey (assuming equal variances) or Games-Howell (non-equal variances) post-hoc test. In bold, significant associations between variables (p < 0.05)*.

* *p < 0.05 heterozygous vs. major allele*.

# *p < 0.05 heterozygous vs. minor allele*.

**Figure 2:**
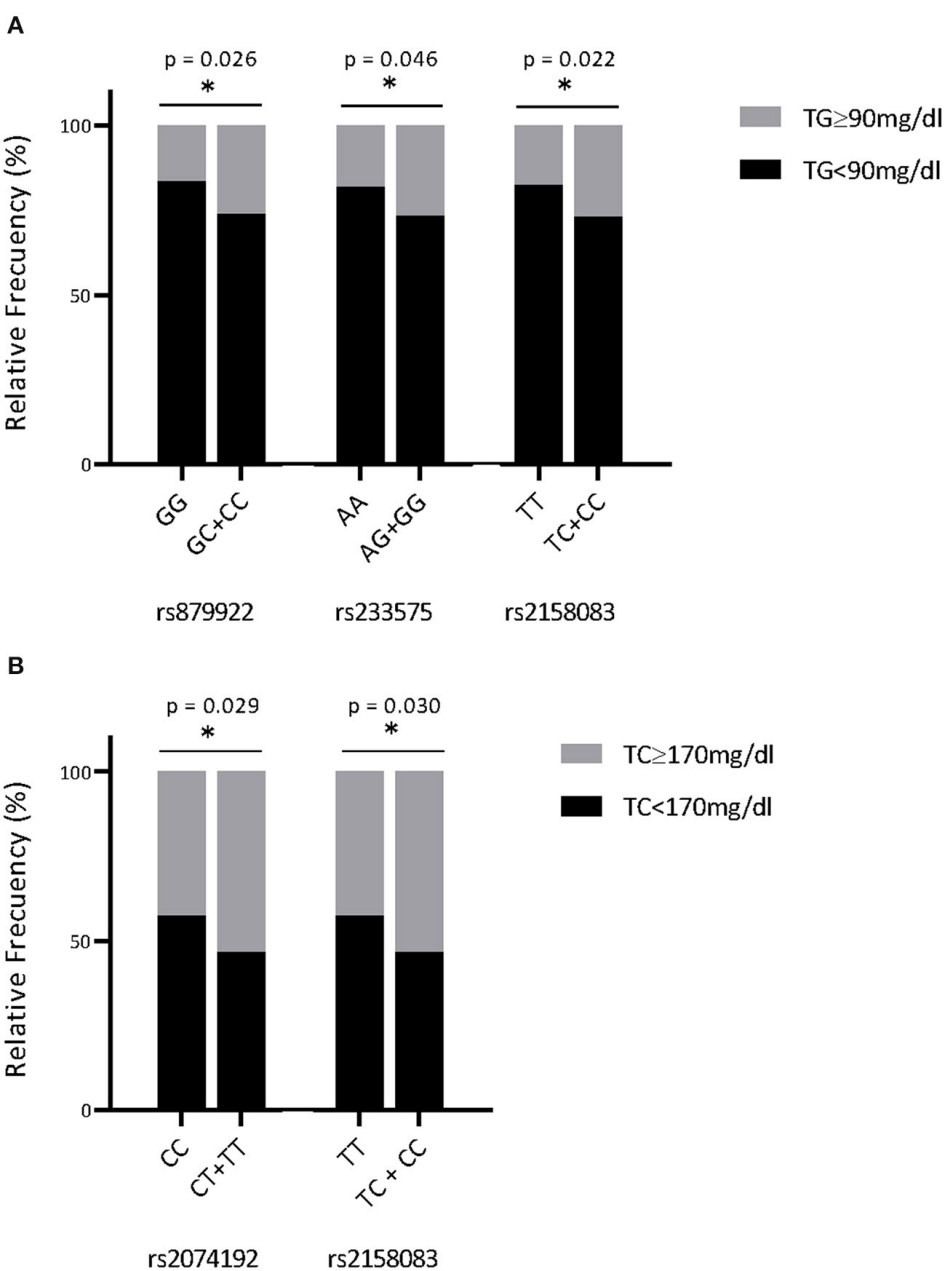
ACE2-SNPs and the risk of elevated TG and TC concentrations. Female adolescents were classified following the TG levels ≥ 90 mg/dL **(A)** or the TC levels ≥ 170 mg/dL **(B)**, and the presence of ACE-SNPs was detected. Data were represented as relative frequencies (%), and associations between variables were analyzed by the Pearson's chi-square test, followed by logistic models. *p < 0.05 minor vs. major allele.

### Association Between *ACE2*-haplotypes and Overweight/Obesity and TG Levels

Since rs879922, rs233575, rs2074192, and rs2158083 were related to overweight/obesity and lipid alterations in females, we examined whether combinations of these SNPs might also associate with higher risk of both pathologies. In particular, the haplotype composed by the minor alleles of rs879922, rs233575, and rs2158083 (C-G-C) was overrepresented in girls (34%) and linked to significantly higher BMI [coef. 0.01 (0.001, 0.019; 95% CI), *p* = 0.038], in comparison with the haplotype composed by the major alleles (G-A-T) (Table 5). The C-G-C haplotype was also associated with the presence of overweight/obesity [OR 1.41 (1.01, 1.97; 95% CI), *p* = 0.044], and elevated plasma TG [coef. 0.023 (0.002, 0.044; 95% CI), *p* = 0.031] and TG/HDL ratio [(coef. 0.031 (0.001, 0.061; 95% CI), *p* = 0.045]. Interestingly, after adjusting by BMI, the C-G-C haplotype maintained its association with higher TG levels [coef. 0.023 (0.002, 0.044 95% CI), *p* = 0.034] (Table 5). On the other hand, the haplotype composed by the minor alleles of rs22074192 and rs2158083 (C-C) was overrepresented in 37% subjects but did not significantly associate with either BMI or lipid levels (not shown). Thus, the C-G-C haplotype of rs879922, rs233575, and rs2158083 SNPs could be considered a risk marker for obesity and dyslipemia in females.

**Table 5:** *ACE2*-haplotypes and the risk of obesity and elevated TG levels.

** 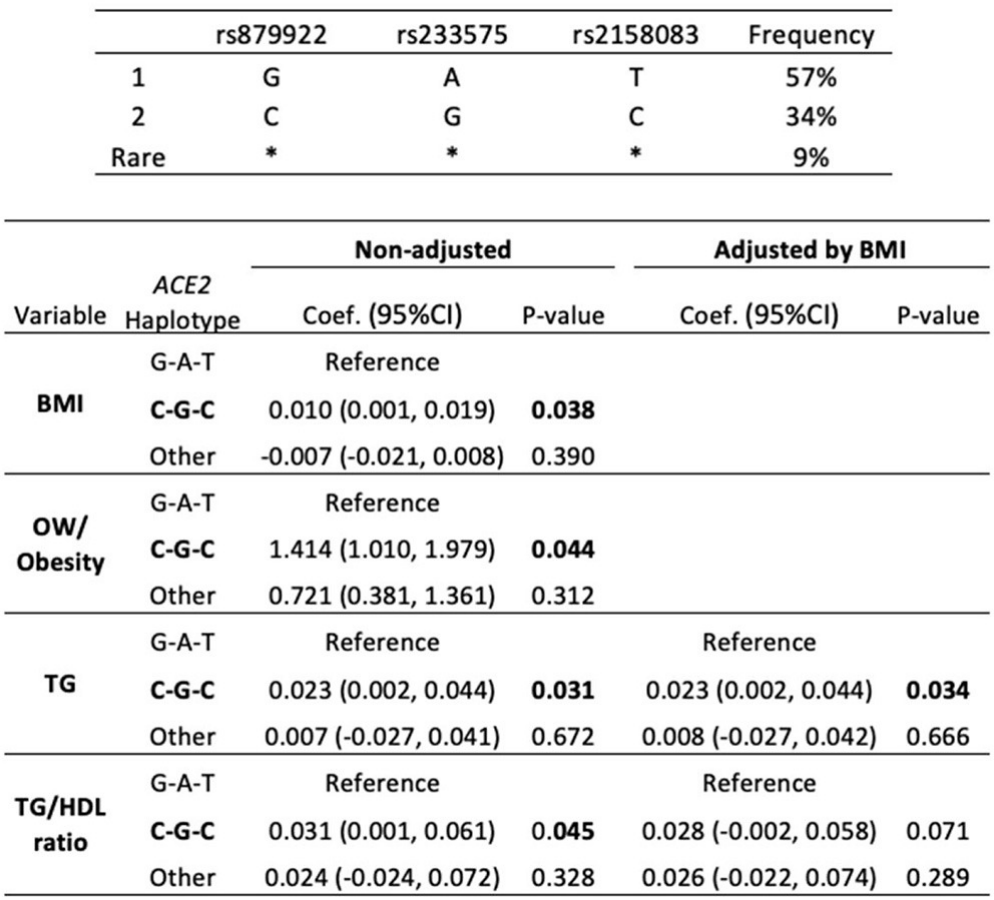 **

*The three ACE2-SNPs (rs879922, rs233575, and rs2158083) related to OW/obesity and elevated plasma lipids in females were analyzed as haplotype. The frequency of minor C-G-C and major G-A-T alleles (top) and their associations with BMI, OW/obesity, TG, and the TG/HDL ratio (bottom) were included. BMI, TG y TG/HDL are shown as coefficient of linear regression. OW/Obesity is shown as Odds ratio of logistic regression*.

*In bold, statistically significant data (p < 0.05)*.

## Discussion

In this work we have analyzed five SNPs in the *ACE2* gene in a general population of apparently healthy adolescents from Spain. The prevalence of these polymorphisms in females and males was comparable to previous findings (12, 22, 23), but the occurrence of some of them was associated with CV risk factors only in girls.

At these ages, females showed lower rates of overweight and obesity, SBP and glycemia than males. Although girls showed higher TC and LDL-C, they enhanced HDL-C, as compensation. They also exhibited lower TG/HDL ratio, which is a predictor of metabolic syndrome and CV diseases (i.e., coronary artery disease or atherosclerosis), and an estimator of LDL-C particle size (20). Thus, theoretically, young girls may be cardio-protected, as previously described (24, 25). Spite of the differences in hormonal regulation between sexes (26), a potential explanation of this cardio-protection could be based on the expression of ACE2 and subsequent effects on the RAAS activity. Since *ACE2* is encoded in chromosome X, and specifically in a region that escape from X-inactivation, *ACE2* may be upregulated in girls. However, this escape gene also shows heterogeneous sex bias that may lead to lower expression (and tissue-specific) and its activity can be regulated by hormone release (27). Nevertheless, the ACE2/Ang (1-7)/MasR activation could preserve pancreas, skeletal muscle, adipose tissue or the CV system from oxidation, inflammation, and fibrosis (28). Moreover, the elevation of ACE2 involve a reduction of Ang-II, which enhances the NADH/NADPH oxidase activity and reduces the nitric oxide-dependent vasodilatation at systemic level (29). Also, Ang-II stimulates cholesterol biosynthesis, overexpresses lipid receptors (i.e., LRP1, LOX-1), facilitates the uptake and oxidation of LDL-C, and induces pro-inflammatory matrix proteins and matrix-degrading enzymes (i.e., MCP-1, IL-18, PAI-1) (30, 31). In addition, ACE2 may defend the CV system by direct actions. ACE2 increases mitochondrial ATP production and reduces pro-oxidant factors such as NOX4. It also ameliorates lipid deposition and endoplasmic reticule stress via GRP78/eIF2α/XBP-1/ATF4/CHOP expression (32).

Importantly, a reduction of ACE2 levels and subsequent Ang (1-7)-MasR signaling can promote CV failures (33). ACE2 knockout mice showed increased lipid accumulation, ER stress and mitochondrial dysfunction in skeletal muscle, and overexpression of ACE2 reduced these responses and improved glucose and lipid metabolism through the IKKβ/NFkB/IRS-1 pathway (32, 34). Similarly, MasR knockout exhibited dyslipemia and insulin resistance, along with decreased adiponectin secretion and glucose uptake (35). Furthermore, during hypertension or diabetes, a deficiency of ACE2 and increased ACE/ACE2 ratio have been noted (36), and cardioprotective treatments with ACE inhibitors and Ang-II receptor blockers exert their effects partly by increasing ACE2 levels (37). In this regard, we propose that in those girls who carried specific intergenic ACE2 SNPs, the integrity, activity and/or stability of the ACE2 RNA-messenger and resultant protein could have been affected (38, 39), and Ang-II signaling may overcome those of ACE2/Ang (1-7)/MasR. In fact, the existence of rs879922, rs233575, and rs2158083 SNPs of *ACE2* was associated with overweight/obesity and elevated plasma TG in Spanish girls. The rs879922 and rs233575 polymorphisms were also related to upper TG/HDL-C ratio, and the rs2074192 and rs2158083 with higher TC levels. Reinforcing our findings, we observed that the C-G-C haplotype of rs879922, rs233575, and rs2158083 was associated with excessive weight and elevated TG levels. Previous data on *ACE2* haplotypes linked higher risk of cardiac hypertrophy with minor alleles of rs2074192 and rs2106809 in females (10) or with those of rs2106809 and rs6632677 in males (40).

Little is known about rs2158083 and rs233575. The former is encoded in *ACE2*'s intron 4 and was previously associated with higher blood pressure (12). Similarly, rs233575 (intron 16) was related to higher blood pressure in adolescents and its interaction with obesity was present only in females (13). It was also linked to increased LDL-C (≥1.8 mmol/L) (8) and T2DM (11) and associated with higher LVMI (41). The rs233575 SNP may alter binding of regulatory factors such as RNA-binding proteins involved in splicing (e.g., ARID5A), which is stimulated under inflammation (42). Moreover, rs879922 (intron 11) can be considered a significant quantitative trait locus for essential hypertension in female Chinese, where it also associated with increased TC (≥5.2 mmol/L) and LDL-C (≥1.8 mmol/L) (8). rs879922 was linked with T2DM (11) and cardiac hypertrophy (41), and importantly, the relative expression of *ACE2* was significantly lower in subjects with the CC genotype of rs879922 compared with those carrying the GG/CG genotype (43). Finally, rs2074192 (intron 16) has been associated with CV risk (i.e., LVH, carotid arteriosclerosis stenosis, microalbuminuria), retinopathy in T2DM individuals, hypertension, and cardiac hypertrophy (10–12). rs2074192 was also linked to increased TC (≥5.2 mmol/L) in hypertensive population (8), and neonates with rs2074192 were more likely to be born as small for gestational age babies, which contribute to metabolic syndrome and CV diseases in later life (44). Interestingly, rs2074192 was associated with reduced circulating Ang (1-7) levels (22), and in COVID-19 patients, rs2074192 correlated to more severe outcomes (i.e., bilateral pneumonia, dyspnea, high fever) (13, 45).

This study lays the groundwork for subsequent trials in patients and experimental animals. The *ACE2*-SNPs, rs879922, rs233575, rs2158083, and rs2074192, could help to classify girls at risk of obesity and dyslipidemia in adulthood. The presence of these SNPs should be confirmed in populations of adult women with obesity, dyslipidemia and/or cardiovascular diseases. Also, this work suggests that variability of the *ACE2* gene could predispose patients to certain metabolic or cardiovascular conditions that are key in the evolution of other diseases (i.e., COVID-19). Furthermore, these *ACE2*-mutations might be generated in rodent models with/without obesity and cardiovascular failures in order to characterize atherogenic responses under *ACE2* modulation.

## Limitations of the Study

At these ages, girls and boys may produce different sexual hormones and factors, which can influence on the lipid profile. Thus, their puberal stage may add important information to explain differences between sex. Also, the precise biological mechanism and other possible factors underlying the association of the *ACE2* gene with overweight/obesity or plasma lipids elevation remain to be clarified. Finally, functional investigations of the association between C-G-C haplotype with overweight/obesity or TG elevation could be required.

## Conclusion

The ACE2 gene may become a double-sword factor for CV protection, at least in women. Those female adolescents who carry the wild-type *ACE2* gene may be somehow protected from increased adiposity and hyperlipidemia. However, Spanish girls carrying the rare alleles of the *ACE2*-SNPs rs879922, rs233575, rs2158083, or rs2074192 may be vulnerable to future obesity and CV injuries. The haplotype rs879922-rs233575-rs2158083 may be considered a valid biomarker for both pathologies. Therefore, these *ACE2*-SNPs might be addressed for therapeutic and prognostic purposes against CV diseases. In this sense, new studies might evaluate their role as predictors of worse evolution in COVID-19 subjects, where CV homeostasis may be seriously damaged.

## Data Availability Statement

The data presented in the study are deposited in the dbSNP repository, submission batch ID 1063361 (dbSNP Build ID: B156).

## Ethics Statement

The study protocol was approved by the Ethics Committee of Clinical Investigation of the Fundación Jiménez Díaz (Code number: PIC016-2019 FJD). Written informed consent to participate in this study was provided by the participants' legal guardian/next of kin.

## Author Contributions

JL-C, CV-V, and IP-N quantified the gene expression of *ACE2*-SNPs and analyze its associations with clinical variables. IM-F performed the statistic studies. LS-G, OL, and CG designed and wrote the manuscript. All authors participate in the discussion of the work.

## Funding

This research was funded by SPACE2-CV-COVID-CM grant from REACT-EU, Comunidad de Madrid and European Regional Development Fund, and by the Fondo de Investigación Sanitaria-IS. Carlos III (ref.: PI18/01016 and PI20/00923), Biobank (ref.: FEDERRD09/0076/00101), and Ciberdem (ref.: CB07/08/2007). JL-C was granted by FPI contract from Universidad Autónoma, Madrid.

## Conflict of Interest

The authors declare that the research was conducted in the absence of any commercial or financial relationships that could be construed as a potential conflict of interest.

## Publisher's Note

All claims expressed in this article are solely those of the authors and do not necessarily represent those of their affiliated organizations, or those of the publisher, the editors and the reviewers. Any product that may be evaluated in this article, or claim that may be made by its manufacturer, is not guaranteed or endorsed by the publisher.

## Abbreviations

ACE: angiotensin convertase enzyme
ACE2: Angiotensin converting enzyme 2
Ang (1-7): angiotensin 1–7
Ang (1-9): angiotensin 1–9
Ang I: angiotensin-I
Ang-II: angiotensin-II
ARID5A: AT-Rich Interaction Domain 5A
AT1R: angiotensin II receptor type 1
AT2R: angiotensin II receptor type 2
ATP: Adenosine triphosphate
BMI: body mass index
COVID: Coronavirus disease
CV: cardiovascular
DBP: diastolic blood preassure
GRP78/eIF2α/XBP-1/ATF4/CHOP, Glucose-Regulated Protein: 78-KD/Eukaryotic Initiation Factor 2/X-box binding protein 1/C/EBP Homologous Protein
HDL-C: high-density lipoprotein-cholesterol
HOMA-IR: homeostatic model assessment of insulin resistance
IKKβ/NFkB/IRS-1: IκB Kinase-beta/nuclear factor-kappa B/insulin receptor substrate 1
IL-18: interleukin-18
LDL-C: low-density lipoprotein-cholesterol
LOX-1: lectin-like OxLDL receptor 1
LRP1: LDL Receptor Related Protein 1
LVMI: left ventricular mass index
MasR: Mas receptor
MCP-1: Monocyte chemoattractant protein-1
NAD(P)H: nicotinamide adenine dinucleotide (phosphate) hydrogen
NOX4: NADPH oxidase 4
NW: normal weight
OR: odds ratio
OW: overweight
PAI-1: Plasminogen activator inhibitor-1
RAAS: Renin-Angiotensin-Aldosterone system
SBP: systolic blood pressure
SNP: single nucleotide polymorphism
T2DM: type-2 diabetes mellitus
TC: total cholesterol
TG: triglycerides.
