# Supplementary figures and images for: Association of ACE2 Polymorphisms and Derived Haplotypes With Obesity and Hyperlipidemia in Female Spanish Adolescents
Source: Front Cardiovasc Med. 2022 May 2;9:888830. doi: 10.3389/fcvm.2022.888830 (PMC9108422; doi:10.3389/fcvm.2022.888830)

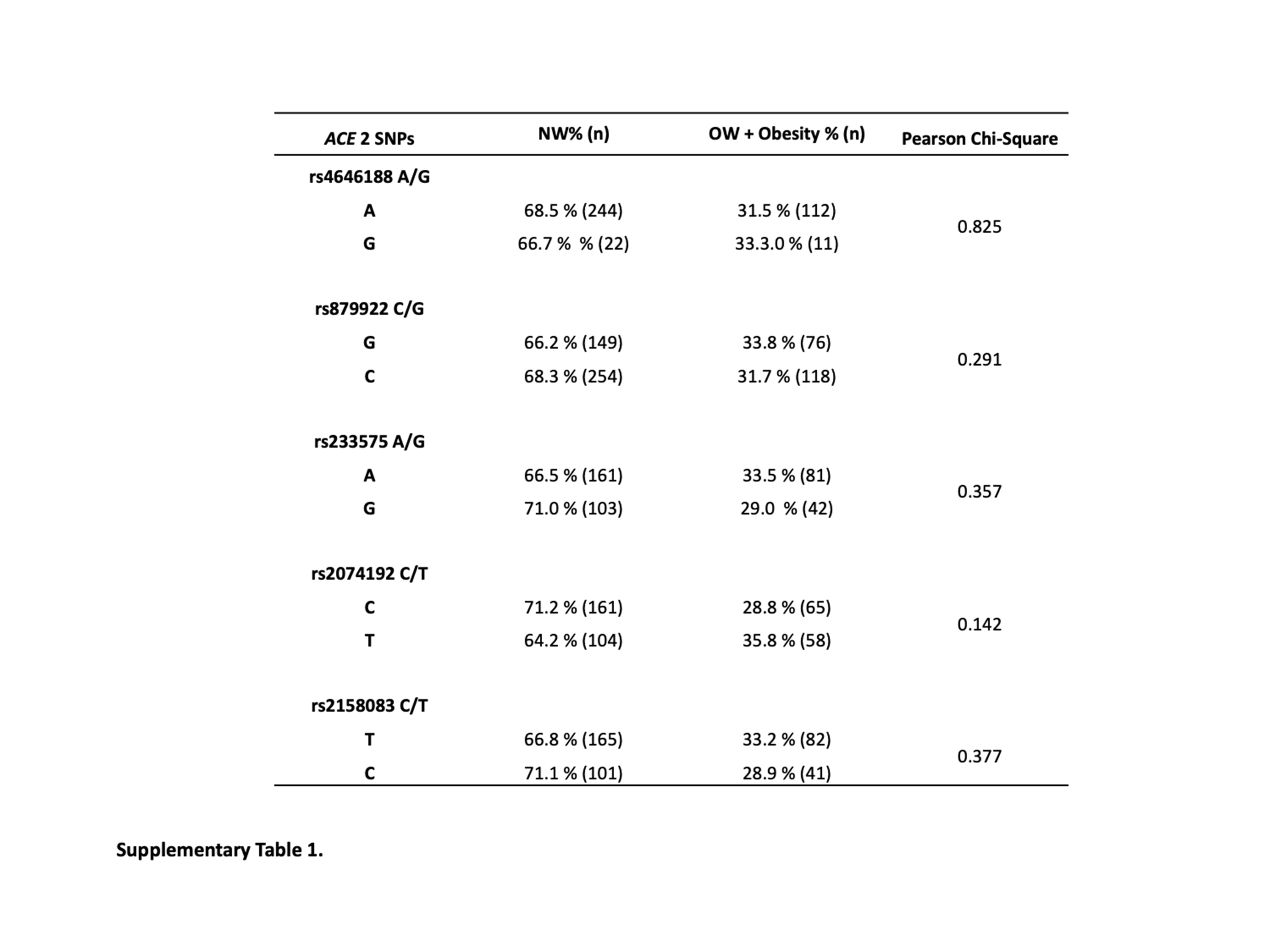

Supplement: Supplementary file 1 [file Image_1.tiff]
